# Correction

**DOI:** 10.1093/immadv/ltac018

**Published:** 2022-08-11

Upon the original publication of the below listed articles, the Data Availability statement was inadvertently omitted. This correction has been published to address the omission and note that the statements have been instated for the following papers:

- Emily Gwyer Findlay, Greg Sutton, Gwo-Tzer Ho, BSI Inflammation Affinity Group Trialswatch Team, The MARVEL trial: a phase 2b randomised placebo-controlled trial of oral MitoQ in moderate ulcerative colitis, *Immunotherapy Advances*, Volume 1, Issue 1, January 2021, ltaa002, https://doi.org/10.1093/immadv/ltaa002
- Dharanidharan Ramamurthy, Trishana Nundalall, Sanele Cingo, Neelakshi Mungra, Maryam Karaan, Krupa Naran, Stefan Barth, Recent advances in immunotherapies against infectious diseases, *Immunotherapy Advances*, Volume 1, Issue 1, January 2021, ltaa007, https://doi.org/10.1093/immadv/ltaa007
- Wilson Savino, Beatriz Chaves, Adriana Cesar Bonomo, Vinicius Cotta-de-Almeida, Integrin-directed antibody-based immunotherapy: focus on VLA-4, *Immunotherapy Advances*, Volume 1, Issue 1, January 2021, ltab002, https://doi.org/10.1093/immadv/ltab002
- Laura J Pallett, Sarah Dimeloe, Linda V Sinclair, Adam J Byrne, Anna Schurich, A glutamine ‘tug-of-war’: targets to manipulate glutamine metabolism for cancer immunotherapy, *Immunotherapy Advances*, Volume 1, Issue 1, January 2021, ltab010, https://doi.org/10.1093/immadv/ltab010

